# Supplementary material for: Unveiling the Bioactive Potential of the Invasive Jellyfish Phyllorhiza punctata Through Integrative Transcriptomic and Proteomic Analyses
Source: Biomolecules. 2025 Aug 4;15(8):1121. doi: 10.3390/biom15081121 (PMC12383608; doi:10.3390/biom15081121)
Supplement: Supplementary file 1 [file biomolecules-15-01121-s001.zip › Suplementary Material/FileS3/PEPFOLD4/XP_065069392_8606/clusters.html]

 PEP-FOLD clusters


Download archive of all models  here   

|  |  |  |
| --- | --- | --- |
| # | file | sOPEP |
| # cluster 1 - Size: 1 |  |  |
|  | XP\_065069392\_8606-model1 | -41.9998 |
| # cluster 2 - Size: 7 |  |  |
|  | XP\_065069392\_8606-model2 | -39.4163 |
|  | XP\_065069392\_8606-model2.1 | -34.254 |
|  | XP\_065069392\_8606-model2.2 | -33.3064 |
|  | XP\_065069392\_8606-model2.3 | -32.4685 |
|  | XP\_065069392\_8606-model2.4 | -32.3531 |
|  | XP\_065069392\_8606-model2.5 | -31.6335 |
|  | XP\_065069392\_8606-model2.6 | -31.2461 |
| # cluster 3 - Size: 9 |  |  |
|  | XP\_065069392\_8606-model3 | -38.5497 |
|  | XP\_065069392\_8606-model3.1 | -36.875 |
|  | XP\_065069392\_8606-model3.2 | -36.0014 |
|  | XP\_065069392\_8606-model3.3 | -35.8063 |
|  | XP\_065069392\_8606-model3.4 | -35.7125 |
|  | XP\_065069392\_8606-model3.5 | -35.3879 |
|  | XP\_065069392\_8606-model3.6 | -35.3422 |
|  | XP\_065069392\_8606-model3.7 | -34.7461 |
|  | XP\_065069392\_8606-model3.8 | -34.4437 |
| # cluster 4 - Size: 1 |  |  |
|  | XP\_065069392\_8606-model4 | -37.7652 |
| # cluster 5 - Size: 3 |  |  |
|  | XP\_065069392\_8606-model5 | -37.6883 |
|  | XP\_065069392\_8606-model5.1 | -34.7411 |
|  | XP\_065069392\_8606-model5.2 | -34.1664 |
| # cluster 6 - Size: 3 |  |  |
|  | XP\_065069392\_8606-model6 | -37.3517 |
|  | XP\_065069392\_8606-model6.1 | -33.2101 |
|  | XP\_065069392\_8606-model6.2 | -31.8473 |
| # cluster 7 - Size: 2 |  |  |
|  | XP\_065069392\_8606-model7 | -37.201 |
|  | XP\_065069392\_8606-model7.1 | -32.2154 |
| # cluster 8 - Size: 4 |  |  |
|  | XP\_065069392\_8606-model8 | -36.9587 |
|  | XP\_065069392\_8606-model8.1 | -35.2337 |
|  | XP\_065069392\_8606-model8.2 | -34.9797 |
|  | XP\_065069392\_8606-model8.3 | -32.0614 |
| # cluster 9 - Size: 1 |  |  |
|  | XP\_065069392\_8606-model9 | -36.8617 |
| # cluster 10 - Size: 1 |  |  |
|  | XP\_065069392\_8606-model10 | -36.7895 |
| # cluster 11 - Size: 5 |  |  |
|  | XP\_065069392\_8606-model11 | -36.7389 |
|  | XP\_065069392\_8606-model11.1 | -34.2281 |
|  | XP\_065069392\_8606-model11.2 | -34.1911 |
|  | XP\_065069392\_8606-model11.3 | -32.2063 |
|  | XP\_065069392\_8606-model11.4 | -31.9879 |
| # cluster 12 - Size: 1 |  |  |
|  | XP\_065069392\_8606-model12 | -36.6037 |
| # cluster 13 - Size: 4 |  |  |
|  | XP\_065069392\_8606-model13 | -36.4383 |
|  | XP\_065069392\_8606-model13.1 | -36.0676 |
|  | XP\_065069392\_8606-model13.2 | -35.3419 |
|  | XP\_065069392\_8606-model13.3 | -33.8116 |
| # cluster 14 - Size: 4 |  |  |
|  | XP\_065069392\_8606-model14 | -36.3711 |
|  | XP\_065069392\_8606-model14.1 | -34.9113 |
|  | XP\_065069392\_8606-model14.2 | -34.7501 |
|  | XP\_065069392\_8606-model14.3 | -34.7043 |
| # cluster 15 - Size: 1 |  |  |
|  | XP\_065069392\_8606-model15 | -36.3645 |
| # cluster 16 - Size: 1 |  |  |
|  | XP\_065069392\_8606-model16 | -36.2886 |
| # cluster 17 - Size: 2 |  |  |
|  | XP\_065069392\_8606-model17 | -36.2881 |
|  | XP\_065069392\_8606-model17.1 | -35.9589 |
| # cluster 18 - Size: 1 |  |  |
|  | XP\_065069392\_8606-model18 | -36.0776 |
| # cluster 19 - Size: 2 |  |  |
|  | XP\_065069392\_8606-model19 | -35.951 |
|  | XP\_065069392\_8606-model19.1 | -35.0546 |
| # cluster 20 - Size: 1 |  |  |
|  | XP\_065069392\_8606-model20 | -35.8598 |
| # cluster 21 - Size: 2 |  |  |
|  | XP\_065069392\_8606-model21 | -35.7717 |
|  | XP\_065069392\_8606-model21.1 | -34.2838 |
| # cluster 22 - Size: 1 |  |  |
|  | XP\_065069392\_8606-model22 | -35.3977 |
| # cluster 23 - Size: 2 |  |  |
|  | XP\_065069392\_8606-model23 | -35.3891 |
|  | XP\_065069392\_8606-model23.1 | -33.9717 |
| # cluster 24 - Size: 1 |  |  |
|  | XP\_065069392\_8606-model24 | -35.339 |
| # cluster 25 - Size: 1 |  |  |
|  | XP\_065069392\_8606-model25 | -35.3241 |
| # cluster 26 - Size: 2 |  |  |
|  | XP\_065069392\_8606-model26 | -35.2057 |
|  | XP\_065069392\_8606-model26.1 | -32.5028 |
| # cluster 27 - Size: 1 |  |  |
|  | XP\_065069392\_8606-model27 | -35.1331 |
| # cluster 28 - Size: 1 |  |  |
|  | XP\_065069392\_8606-model28 | -34.9327 |
| # cluster 29 - Size: 1 |  |  |
|  | XP\_065069392\_8606-model29 | -34.68 |
| # cluster 30 - Size: 1 |  |  |
|  | XP\_065069392\_8606-model30 | -34.6095 |
| # cluster 31 - Size: 1 |  |  |
|  | XP\_065069392\_8606-model31 | -34.2081 |
| # cluster 32 - Size: 1 |  |  |
|  | XP\_065069392\_8606-model32 | -34.0628 |
| # cluster 33 - Size: 2 |  |  |
|  | XP\_065069392\_8606-model33 | -33.8496 |
|  | XP\_065069392\_8606-model33.1 | -33.3979 |
| # cluster 34 - Size: 1 |  |  |
|  | XP\_065069392\_8606-model34 | -33.8345 |
| # cluster 35 - Size: 1 |  |  |
|  | XP\_065069392\_8606-model35 | -33.7436 |
| # cluster 36 - Size: 1 |  |  |
|  | XP\_065069392\_8606-model36 | -33.7271 |
| # cluster 37 - Size: 8 |  |  |
|  | XP\_065069392\_8606-model37 | -33.4173 |
|  | XP\_065069392\_8606-model37.1 | -33.0952 |
|  | XP\_065069392\_8606-model37.2 | -32.9285 |
|  | XP\_065069392\_8606-model37.3 | -32.6677 |
|  | XP\_065069392\_8606-model37.4 | -32.346 |
|  | XP\_065069392\_8606-model37.5 | -31.8863 |
|  | XP\_065069392\_8606-model37.6 | -31.7082 |
|  | XP\_065069392\_8606-model37.7 | -31.3944 |
| # cluster 38 - Size: 2 |  |  |
|  | XP\_065069392\_8606-model38 | -33.3076 |
|  | XP\_065069392\_8606-model38.1 | -32.7531 |
| # cluster 39 - Size: 1 |  |  |
|  | XP\_065069392\_8606-model39 | -33.1177 |
| # cluster 40 - Size: 7 |  |  |
|  | XP\_065069392\_8606-model40 | -32.4995 |
|  | XP\_065069392\_8606-model40.1 | -31.9497 |
|  | XP\_065069392\_8606-model40.2 | -31.8001 |
|  | XP\_065069392\_8606-model40.3 | -31.6946 |
|  | XP\_065069392\_8606-model40.4 | -31.3548 |
|  | XP\_065069392\_8606-model40.5 | -30.8695 |
|  | XP\_065069392\_8606-model40.6 | -29.9966 |
| # cluster 41 - Size: 1 |  |  |
|  | XP\_065069392\_8606-model41 | -32.4942 |
| # cluster 42 - Size: 2 |  |  |
|  | XP\_065069392\_8606-model42 | -31.8574 |
|  | XP\_065069392\_8606-model42.1 | -30.1372 |
| # cluster 43 - Size: 2 |  |  |
|  | XP\_065069392\_8606-model43 | -31.3211 |
|  | XP\_065069392\_8606-model43.1 | -31.267 |
| # cluster 44 - Size: 1 |  |  |
|  | XP\_065069392\_8606-model44 | -30.7745 |
| # cluster 45 - Size: 1 |  |  |
|  | XP\_065069392\_8606-model45 | -29.0162 |
| # cluster 46 - Size: 1 |  |  |
|  | XP\_065069392\_8606-model46 | -23.6921 |
|  |  |  |
